# Artificial intelligence in healthcare in Latin America and Colombia: bridging gaps and leveraging opportunities

**DOI:** 10.15649/cuidarte.6342

**Published:** 2026-08-14

**Authors:** Santiago Sierra-Castillo, David Aristizábal-Colorado, Martha Eugenia Reyes-Sarmiento

**Affiliations:** 1 Departamento de epidemiologia, Universidad CES, Medellín, Antioquia. Escuela de Administración, EAFIT, Medellín, Colombia. E-mail: santiagosierrascc@gmail.com Universidad CES Medellin Colombia santiagosierrascc@gmail.com; 2 Departamento de Cardiología, Universidad de Antioquia. Medellín, Antioquia. Grupo de investigación en Medicina Interna (GIMI1), Universidad Libre. Cali, Colombia. E-mail: dvdrstzbl@gmail.com Universidad Libre Cali Colombia dvdrstzbl@gmail.com; 3 Directora de Área de Organización, Dirección y Estrategia EAFIT, Medellín, Antioquia. E-mail: mreyess@eafit.edu.co Dirección y Estrategia EAFIT Medellin Colombia mreyess@eafit.edu.co

**Artificial intelligence: An opportunity amid structural gaps**

Artificial intelligence (AI) is rapidly transforming multiple fields, particularly medicine and the health sciences, not only as a technological innovation but also as a potential shift in how clinical decisions are made, healthcare services are organized, and health systems are structured^1^. From diagnostic algorithms and predictive models to the recent emergence of generative models and generative AI tools, AI has evolved from isolated applications into technologies capable of reshaping clinical care processes as well as operational and administrative functions related to healthcare delivery^2^-^5^. The exponential advancement of this technology has generated considerable expectations regarding its potential to improve quality, efficiency, and access to healthcare, while also raising important concerns about safety, equity, governance, and accountability^6^.

As health systems begin to adopt and explore the use of AI, it is becoming increasingly clear that the analysis of these technologies can no longer focus solely on the technical capabilities of algorithms. The more critical question is whether AI can be effectively integrated to strengthen health systems and how this can be achieved, given its potential to help reduce structural gaps. However, inappropriate implementation carries the risk of reproducing and even amplifying pre-existing inequalities, particularly in settings such as Latin American countries, where structural barriers remain pervasive, including those observed in Colombia^7^,^8^.

This question is particularly important in Latin America, where health systems face institutional fragmentation, health workforce shortages, financial constraints, and persistent gaps in access to care^9^,^10^. In this context, AI represents not only a frontier technological innovation but also a potential tool for expanding capacity in areas where structural limitations currently exist^10^.

Several studies have demonstrated the utility of machine learning–based tools for specific diagnostic tasks, clinical risk prediction, and support for repetitive processes^8^,^11^,^12^. More recently, the expansion of generative AI models has created new opportunities in clinical documentation, medical education, conversational support, and the analysis of complex information. These applications are enabled by advanced deep learning capabilities, the processing of large volumes of data, and the development of models capable of generating content and supporting decision-making processes in complex environments^13^,^14^.

However, technological potential alone does not guarantee improvements in health outcomes. The implementation of AI in healthcare is, fundamentally, a sociotechnical challenge^3^,^10^,^15^. The performance of AI models is important, but so are data quality, regulatory frameworks, institutional trust, and the readiness of health systems and their stakeholders to adopt these technologies^4^,^10^-^12^,^16^. Indeed, the healthcare sector is being reshaped by one of the driving forces of the Fourth Industrial Revolution, and whether this transformation unfolds successfully way will depend on those responsible for implementing it: governments and health systems.

**Artificial intelligence at a crossroads: closing or widening gaps**

In Latin America, AI has the potential to become a tool for reducing long-standing inequities or, if implemented without appropriate safeguards, a mechanism capable of deepening them^1^,^17^. This tension reflects one of the major challenges currently facing the integration of these technologies into healthcare. Limitations related to interoperability, digital maturity, data representativeness, and institutional capacity persist and are closely shaped by the heterogeneity of the region’s health systems. In addition, a large proportion of technological developments originate outside Latin America, raising important questions about their external validity, technological sovereignty, and the appropriateness of their application in local contexts^10^,^12^,^15^.

The risks are well recognized: algorithmic bias, opacity in decision-making, digital exclusion, systematic errors affecting underrepresented populations, and new forms of technological dependence. However, the opportunities are equally evident: improving access to care, optimizing resource allocation, strengthening continuity of care, and supporting complex clinical decision-making^10^,^15^. In this context, particularly in Latin America, AI could help improve the quality of care, expand access (one of the region’s most persistent challenges), and mitigate the impact of shortages of specialized healthcare personnel, thereby promoting more timely care delivery. These effects could, in turn, reduce the burden on health systems and associated costs, while also influencing social determinants relevant to health across the region^10^,^15^.

**Figure 1:**
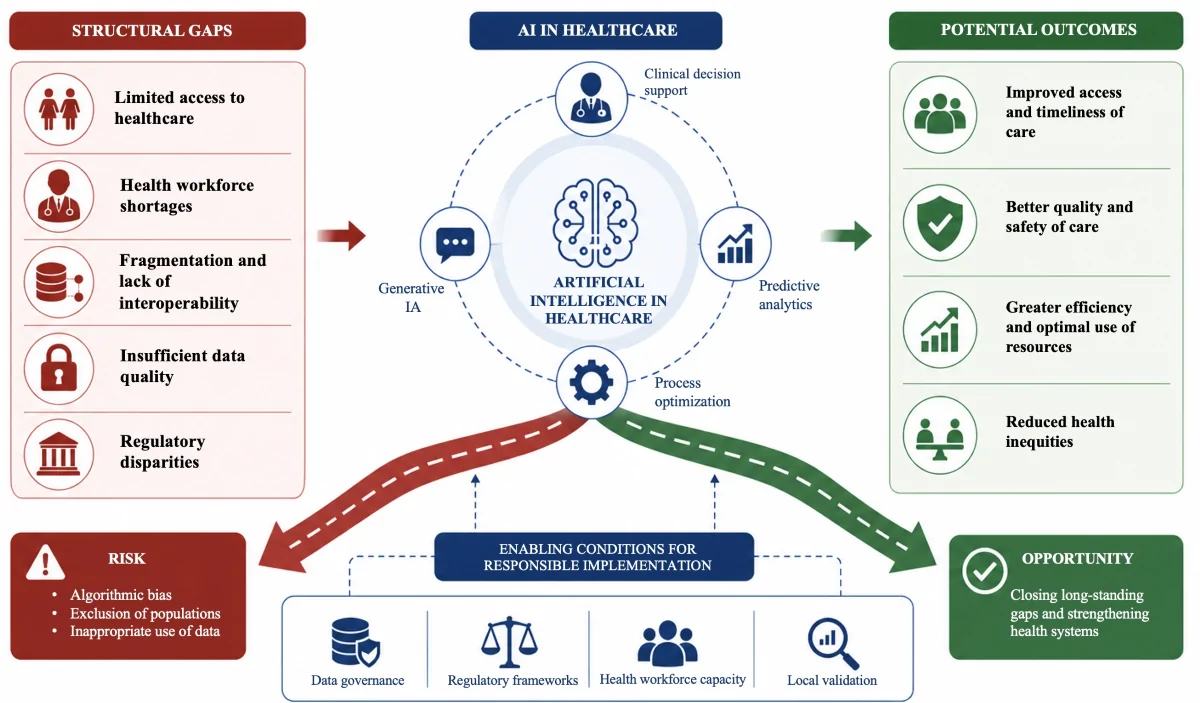
Artificial intelligence as a crossroads for health systems in Latin America

At present, Latin America lacks the computing capacity required to manage large volumes of data, as well as the information technology needed to integrate, adopt, and use AI effectively in the healthcare sector. Across the region, supercomputing infrastructure remains limited compared with that of more technologically advanced regions, restricting the analytical capacity and scalability of these tools. Consequently, without investment in infrastructure, and in policies designed to support its implementation, the potential of AI to transform health systems will remain constrained^10^,^11^,^14^,^15^ Figure 1.

*Artificial intelligence (AI) can either amplify structural gaps or help close them, depending on enabling conditions such as data governance, regulation, ethical frameworks, human resources for health, and local validation. (Figure created with the assistance of generative artificial intelligence [ChatGPT, OpenAI] for visual design and composition).*

**Data governance, ethics, and trust as foundational pillars**

One of the most important debates surrounding the adoption of AI in healthcare concerns data governance. These technologies rely on robust, longitudinal, and representative clinical data, but they also raise critical questions regarding privacy, informed consent, secondary use of data, and digital sovereignty^14^,^15^.

In middle-income countries, these discussions are particularly relevant. It is not only about protecting data but also about determining who controls it, how the value it generates is distributed, and how to avoid new forms of technological dependence^18^,^19^. Equally important is the need to address ethical dimensions such as bias, transparency and accountability. In healthcare, these issues are not ancillary considerations but essential requirements for safe implementation^19^,^20^.

The emergence of generative AI models has made this discussion even more urgent. Although these technologies offer significant opportunities for clinical and administrative support, they also introduce risks related to plausible errors, unsupervised automation, and cognitive dependence^15^. This reinforces the need for innovation and caution to advance hand in hand.

**Colombia as an opportunity for responsible innovation**

Colombia reflects many of these regional tensions. Although the country has made progress in digitalization and innovation, barriers related to interoperability, fragmentation of clinical information, nascent regulatory frameworks, and heterogeneous institutional capacities remain^16^.

Recent evidence from Colombian institutions indicates that patients, healthcare administrators, and engineers simultaneously recognize both the transformative potential of AI and the challenges associated with its implementation, including data quality, sustainability, regulatory frameworks, and institutional trust^14^,^16^,^20^.

This finding suggests that the discussion is not merely technological but also organizational and political. For this very reason, Colombia could make a significant contribution to developing regional models for the responsible implementation of AI in healthcare.

Colombia is making progress toward the integration of AI within the public sector, where initiatives have been put forward to explore its applications across different sectors, including healthcare. However, these initiatives, promoted by the institutions responsible for health regulation, remain limited and lack a clear roadmap for implementation. In this context, the main challenges can be grouped into several key dimensions: data quality, contextual understanding, regulatory frameworks, education and resistance to change, research and development, financial resources, infrastructure and connectivity, as well as scalability^16^.

**Five priorities for a regional agenda**

In light of this landscape, a regional agenda for AI in healthcare should prioritize at least five key areas^10^,^14^,^15^.

1. Strengthen digital infrastructure and interoperability as prerequisites for scalability.

2. Promote local clinical validation and regional evidence generation, while avoiding the uncritical adoption of externally developed solutions.

3. Develop risk-based regulatory frameworks capable of balancing innovation with safety and ethical oversight.

4. Invest in health workforce capacity and artificial intelligence literacy as prerequisites for safe adoption.

5. Make equity and public value creation the guiding principles for impact assessment

These priorities do not represent an exclusively technological agenda but rather a strategy for directing innovation toward strengthening health systems.

**From technological adoption to value creation**

Perhaps one of the most important contributions of AI will not be to automate medicine but to help humanize it. Reducing administrative burden, strengthening continuity of care, and freeing up clinical time for the physician-patient relationship may be among its most valuable applications^14^.

For Latin America, this opportunity carries important strategic implications. The region can either limit itself to adopting technologies designed around external priorities or develop its own models of responsible innovation aimed at addressing local structural challenges^7^,^10^,^15^.

Artificial intelligence will not, by itself, resolve the region’s structural challenges. However, if implemented appropriately, it can become a powerful tool for addressing them. The real challenge is not technological but political, ethical, and institutional^14^,^19^.

Ultimately, the question is not whether artificial intelligence will reach Latin American health systems, but whether it will be used to close long-standing gaps or to deepen them. Its impact will depend not only on its technical capabilities but also on the conditions under which it is implemented: data governance, the robustness of regulatory frameworks, validation in local contexts, and the capacity of health systems to integrate it effectively.

In a region marked by structural limitations in access, financing, and institutional capacity, artificial intelligence represents both an opportunity and a risk. Its implementation can contribute to improving the quality of care, optimizing resource use, and expanding access to healthcare; however, it can also reproduce inequities if it is not guided by a framework grounded in equity and public value. In this regard, advancing toward the responsible adoption of AI requires strengthening institutional capacities, investing in infrastructure and health workforce development, and aligning public policies to ensure that these technologies respond to the region’s actual needs.
